# Introduction to the Proceedings of the 12Th Germinal Centre Conference — Thirty Years of Germinal Centre Conferences

**DOI:** 10.1155/1998/63809

**Published:** 1998

**Authors:** P. Nieuwenhuis

**Affiliations:** Department of Histology and Cell Biology University of Groningen Groningen The Netherlands


*Developmental Immunology*, 1998, Vol. 6, pp. 1–2
Reprints available directly from the publisher
Photocopying permitted by license only

© 1998 OPA (Overseas Publishers Association)
N.V. Published by license under the
Harwood Academic Publishers imprint,
part of the Gordon and Breach Publishing Group
Printed in Malaysia

### Introduction to the Proceedings of the 12th Germinal Centre Conference

### Thirty Years of Germinal Centre Conferences

P. NIEUWENHUIS[a]*

[a]*Department of Histology and Cell Biology, University of Groningen, Groningen, The Netherlands*

(*Received 20 August 1996*)

"In Vivo Veritas": Under this motto, the twelfth so-called Germinal Centre Conference was held in Graz, Austria, July 1 to 5, 1996, with Prof. Konrad Schauenstein chairing the organizing committee, with the assistance of Dr. Richard Boyd (Australia).

Despite the progress that is being made using *in vitro* models, some immunologists believe that "the proof of the pudding is in the eating", that is, to understand the physiology of the immune system as it operates under *in vivo* conditions is to understand it all.

In 1966, the first conference on Germinal Centres in Immune Responses was held in Bern, Switzerland, at the initiative of C.C. Congdon (Oak Ridge, TN) and H. Cottier (Bern, Switzerland), and included 57 presentations, "which were discussed at length".

"The range of interest extended from phylogenesis and anatomy to studies on cell proliferation, immunohistochemistry, cancer research and radiobiology.

"The aim of this broad coverage was to combine all available information on the role of germinal centers in immune responses in a single package, instead of leaving it scattered around in reports dealing with divergent immunological problems" (Cottier et al., 1967).

As this meeting, at least to the opinion of those attending, was quite successful, a next meeting was scheduled for 1968 to be held in Padua, Italy, where, in 1604, Hieronymus Fabricius gave the first description of what was later called the Bursa of Fabricius in his honor.

As it had become apparent that Germinal Centers were not the only site of reactivity following antigen administration, the scope of interest broadened and hence the title of these meetings changed into "Conference on Lymphatic Tissue and Germinal Centers in Immune Reactions".

In the index of the *Proceedings of the 3rd GCC*, held in Uppsala, Sweden, in 1970, for the first time "B cells" are mentioned and T cells are still referred to as "Thymus-derived cells", even though in some presentations, the term "T cells" is already being used.

Once these cells and their pivotal role in immune responses had been recognized, a shift to an even

*Corresponding author: Chairman, Scientific Committee for Germinal Centre Conferences.

broader interest in the histophysiology of the immune system was just a small step. However, the original name Germinal Centre Conference lingered on and stuck to these meetings. Essentially, germinal centers symbolize morphological entities that wax and wane in the course of an immune response, the function of which cannot be mimicked in *in vitro* experiments.

Also these meetings function as "germinal centres", stimulating new concepts as only plenary sessions are held and much time is devoted to "social" interactions. From this point of view, it was good to see that so many young scientists participated in the last meeting, indicating that "In Vivo Immunology" is very much alive and kicking!

Already plans for the next meeting are in progress. The 13th GCC will be held in Switzerland, presumably early in July 1999. Dr Marie Kosco-Vilbois (whose address is Glaxo Institute for Molecular Biology, 20C chemin des Aulx, CH-1228 Plan-les-Ouates, Geneva, Switzerland) has kindly undertaken the not so easy task of organizing this meeting to try and surpass previous GCCs.

Finally, on behalf of the Scientific Committee, I would once more like to thank Prof. Konrad Schauenstein and his team for organizing a great meeting, both scientifically as well as socially. Memories of the 12th GCC will linger on in our minds as well as in our hearts. "In Vivo Veritas!"

### *Acknowledgements*

Members of the Scientific Committee included G. Klaus, S. Fossum, B.A. Imhoff, E. Heinen, K. Schauenstein, R.L. Boyd, D.G. Osmond, and G.J. Thorbecke.

### *References*

Cottier H., et al. (1967). Proceedings of a Symposium on Germinal Centers in Immune Responses (Berlin Heidelberg New York, Springer-Verlag), p. v.

